# Defining pediatric traumatic brain injury using International Classification of Diseases Version 10 Codes: A systematic review

**DOI:** 10.1186/s12883-015-0259-7

**Published:** 2015-02-04

**Authors:** Vincy Chan, Pravheen Thurairajah, Angela Colantonio

**Affiliations:** Toronto Rehabilitation Institute, University Health Network, 550 University Avenue, Toronto, ON M5G 2A2 Canada; Rehabilitation Sciences Institute, University of Toronto, 500 University Avenue, Toronto, ON M5G 1V7 Canada; Acquired Brain Injury Research Lab, University of Toronto, 500 University Avenue, Toronto, ON M5G 1V7 Canada

**Keywords:** Coding, International Classification of Diseases, Pediatric brain injury

## Abstract

**Background:**

Although healthcare administrative data are commonly used for traumatic brain injury (TBI) research, there is currently no consensus or consistency on the International Classification of Diseases Version 10 (ICD-10) codes used to define TBI among children and youth internationally. This study systematically reviewed the literature to explore the range of ICD-10 codes that are used to define TBI in this population. The identification of the range of ICD-10 codes to define this population in administrative data is crucial, as it has implications for policy, resource allocation, planning of healthcare services, and prevention strategies.

**Methods:**

The databases MEDLINE, MEDLINE In-Process, Embase, PsychINFO, CINAHL, SPORTDiscus, and Cochrane Database of Systematic Reviews were systematically searched. Grey literature was searched using Grey Matters and Google. Reference lists of included articles were also searched for relevant studies. Two reviewers independently screened all titles and abstracts using pre-defined inclusion and exclusion criteria. A full text screen was conducted on articles that met the first screen inclusion criteria. All full text articles that met the pre-defined inclusion criteria were included for analysis in this systematic review.

**Results:**

A total of 1,326 publications were identified through the predetermined search strategy and 32 articles/reports met all eligibility criteria for inclusion in this review. Five articles specifically examined children and youth aged 19 years or under with TBI. ICD-10 case definitions ranged from the broad injuries to the head codes (ICD-10 S00 to S09) to concussion only (S06.0). There was overwhelming consensus on the inclusion of ICD-10 code S06, intracranial injury, while codes S00 (superficial injury of the head), S03 (dislocation, sprain, and strain of joints and ligaments of head), and S05 (injury of eye and orbit) were only used by articles that examined head injury, none of which specifically examined children and youth.

**Conclusion:**

This review provides evidence for discussion on how best to use ICD codes for different goals. This is an important first step in reaching an appropriate definition and can inform future work on reaching consensus on the ICD-10 codes to define TBI for this vulnerable population.

**Electronic supplementary material:**

The online version of this article (doi:10.1186/s12883-015-0259-7) contains supplementary material, which is available to authorized users.

## Background

Traumatic brain injury (TBI) is the leading cause of death and disability among children and young adults worldwide [1] and can have severe and long term impacts on the individuals’ physical, cognitive, and psychosocial functioning [2]. Globally, TBI affects 10 million individuals annually and it is estimated that by the year 2020, TBI will exceed many diseases as the major cause of death and disability [3]. There is an increasing trend to look to “big data” to advance our understanding of health care conditions including neurological disorders [4]. Healthcare administrative data can provide a rich source for cost effective research and providing common definitions of TBI across jurisdictions can lead to insights in the nature and trends of TBI. There is, however, currently no consensus on the International Classification of Diseases Version 10 (ICD-10) codes to define TBI. The ICD is the “standard diagnostic tool for epidemiology, health management, and clinical purposes” [5]. Currently, the ICD is in its 10th version (ICD-10) and came into use by World Health Organization Member States in 1994. In Canada, the majority of its provinces and territories began using ICD-10 in the year 2001 and by the year 2006, it was completely implemented [6]. It is also currently being used in other countries, including Australia, France, United Kingdom, and Germany [7,8]. Given the widespread use of the ICD-10 codes to identify TBI cases, it is important that an accurate, appropriate, and sensitive case definition is used across countries so we have a common language and measure of the incidence and outcomes of TBI across multi-national jurisdictions.

Current estimates of TBI have varied greatly between countries [9], and a recent opinion piece published in *Nature Reviews Neurology* by Roozenbeek and colleagues suggested that these estimates are likely underestimates due to the variability in coding and case definitions. Further, this review brought attention to the serious consequences of having “inadequate standardization and incomplete capture of data on the incidence and outcome of brain injury”. This has implications for efforts to accurately understand and quantify the burden and outcome of TBI worldwide [10]. Specifically, resource allocation, planning of healthcare services, and prevention strategies are dependent on the cohort of individuals identified by the case definition. If some individuals identified do not truly have a TBI, this can negatively impact prevention efforts and similarly, missing individuals who actually have a TBI makes it difficult to address the needs of the TBI population. Finally, varying case definitions used worldwide makes it difficult to compare across studies and geography.

This systematic review explored the range of ICD-10 codes that are used to define TBI among children and youth aged 19 years and under. This population is at a critical developmental stage of their lives in which adverse events may result in serious negative long-term consequences. In the case of TBI, there are unique features of a pediatric patient [11], including vulnerability of the developing brain and skull, which is not fully formed and thus, makes this population more vulnerable to brain injury and negative long-term consequences [12,13]. These negative outcomes include psychiatric illnesses [14,15], and deficits in cognition, attention, and executive function [16-20]. Finally, the pediatric population may be at risk in abusive situations, including the shaken baby syndrome, which may lead to a TBI [21] and cannot be self reported due to limited communication abilities. Previous studies have identified high rates of TBI in this population. A recent report from the Centre for Diseases Prevention and Control (CDC) in the United States showed that the highest rates of TBI related emergency department (ED) visits from 2002 to 2006 were among children aged 0 to 4 years and older and adolescents aged 15 to 19 years. Approximately half a million ED visits for TBI were made annually by individuals 14 years and under [22]. Between fiscal years 2003/04 and 2009/10 in the province of Ontario in Canada, 36% of all TBI related ED visits and 16% of all TBI related acute care admissions were among children and youth 18 years and under [23].

Current systematic reviews have brought attention to the importance of accurate codes [24,25], however, none specifically examined ICD-10 codes for the children and youth population in detail. Surveillance of TBI in children and youth is crucial and the availability of accurate information is essential for evaluating, planning, and transforming healthcare systems to better address the needs of this population. As such, it is important to identify the range of ICD-10 codes that are used to identify children and youth in order to accurately and appropriately capture this population in healthcare administrative data for research purposes. This systematic review serves to provide a baseline for future work on reaching an appropriate definition and can inform future work on reaching consensus on the ICD-10 codes to define TBI for this vulnerable population.

## Methods/Design

The protocol for this systematic review is published in the journal *Systematic Reviews* [26] and is highlighted below.

### Search strategy

The following databases were searched for relevant articles:

1. MEDLINE (1946 to February Week 2 2013)
2. MEDLINE In-Process (February 19, 2013)
3. Embase (1980 to 2013 Week 07)
4. PsychINFO (1805 to February Week 3 2013)
5. CINAHL (1981 to Present)
6. SPORTDiscus (1800 to Present)
7. Cochrane Database of Systematic Reviews (2005 to January 2013).

SPORTDiscus and Cochrane Database of Systematic Reviews were systematically searched on March 1, 2013 and the rest of the databases were searched between February 21, 2013 and February 28, 2013. Please see Additional file 1 for the search strategy associated with each database. Searches were limited to the year 1992 to present, as the focus of this article is on selecting ICD-10 codes and work on ICD-10 was completed in 1992. Grey literature was also searched using “Grey Matters, A Practical Search Tool for Evidence-Based Medicine” [27] and Google. Please see Additional file 1 for a list of search terms for grey literature. Search terms were also derived using relevant published reviews as guides [24,25,28-32]. The search terms were derived using three concepts – (1) ICD-10, (2) TBI, and (3) children. The concept “ICD-10” included types of research studies (e.g., validation) and sources of data (e.g., administrat* data, medical record*) in which ICD codes are likely to be used. This was done to increase the likelihood that all articles that used ICD-10 codes were captured, should the terms “ICD-10” or “International Classification of Diseases” were not stated in the abstract or listed as a subject heading. The concept “brain injury” purposely included broader terms that may include a TBI, such as “mtbi”, “concuss*”, “brain* adj3 damag*”, etc. Specifically, concussion, acquired TBI, head injury, and head trauma are terms that are sometimes used interchangeably even though they describe different conditions. As such, including articles that examined concussion, acquired TBI, head injury, and head trauma will decrease the chance that some relevant articles will be missed and can also assist in elucidating the codes that are primarily used to identify these various conditions. The final concept “children” included all terms relevant to the children and youth population such as “pediatr*”, “teen*”, and categories in the database in which children and youth may be included (e.g., “young adult/” in Medline and Medline In-Process).

The study selection process along with reasons for exclusion at the full text level was presented via PRISMA study flow diagram. The literature was searched for evidence of a relationship between brain injury and specific conditions to inform the appropriateness of including specific codes in the case definition of TBI in children and youth.

### Study selection

For all databases, two reviewers independently assessed all title and abstracts for fulfillment of predetermined eligibility criteria. A first screen, the title and abstract screen, was conducted on all retrieved articles. Those that passed the first screen had a full text version available and examined concussion, acquired TBI, head injury, or head trauma. Articles that did not examine concussion, acquired TBI, head injury, or head trauma were excluded.

**Figure 1:**
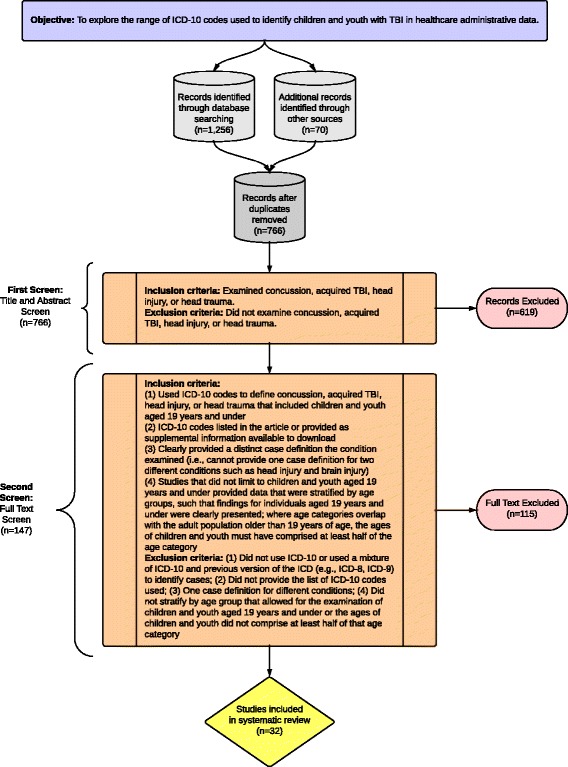
**Description of the study inclusion and exclusion criteria.**

Articles that met any of the above first screen inclusion criteria were included for the second screen, which was a full-text screen. Two reviewers independently assessed all full-text articles for fulfillment of predetermined eligibility criteria. Articles included for the systematic review had to have used ICD-10 codes to define concussion, acquired TBI, head injury, or head trauma that included children and youth aged 19 years and under. These ICD-10 codes must have been listed in the article or have been provided as supplemental information available to download online. The articles had to clearly state the definition of a concussion, TBI, head injury, or head trauma (e.g., articles that provide one definition for TBI and head injury would be excluded, as these are different conditions). Also, studies that did not limit to children and youth aged 19 years and under must have had data that were stratified by age groups, such that findings for individuals aged 19 years or under were clearly presented. Where age categories overlapped with the adult population of older than 19 years of age, children and youth must have comprised at least half of the age category. For example, an article that stratified the data by age groups with the 15 to 24 year olds as the youngest age group would be included because 15 to 19 years of age was 50% of 15 to 24 years age-range. Conversely, an article with the youngest age category of 18 to 25 years of age would be excluded because ages 18 and 19 years were less than 50% of the 18 to 25 years age-range. Please see Figure 1 for a flowchart describing the study selection process.

The reference lists of included full-text articles were also hand-searched on March 15, 2013. An expert in the field of administrative data and TBI was consulted to ensure no additional studies were missed with the use of the above search strategy.

### Data extraction

Study data were abstracted independently by two reviewers and included:

1. Author and publication year
2. ICD-10 codes used to define concussion, acquired TBI, head injury, or head trauma
3. Source of data
4. Year of study
5. Location of study
6. Age of the study population
7. Range of incidence
8. Purpose of the Study.

### Quality assessment

Quality assessment of the codes was determined by whether the ICD-10 codes that were used to define TBI were validated. Findings were categorized into “yes” or “no” (in reports, “no” was replaced by “unclear”, as codes may be validated in other studies but not stated in the reports). Validation of codes is critical, as it provides information on the accuracy of coding and agreement for diagnoses. Given the objective of this review and the importance of validated codes, this quality assessment of the codes was preferred over more standard quality assessment tools.

### Analyses

ICD-10 codes used to define concussion, acquired TBI, head injury, or head trauma were abstracted and each article was categorized by the type of TBI and head injury (mild TBI/concussion, TBI, severe TBI, head injury, and abusive head trauma), purpose of the study (to identify incidence and trends or to identify TBI related deaths), and the target population (≤19 years, ≤25 years, 16+ years, and 0+ years). These categories were created according to results obtained from articles that met the inclusion criteria of this review.

A range of ICD-10 codes used to define TBI in children and youth was identified. Codes that were used consistently among TBI articles and in particular, articles that examined TBI in children and youth aged 19 years or under were suggested for inclusion in the definition of TBI in this population. Where evidence from the literature suggests a relationship between brain injury and the condition the code described, it was also suggested for inclusion in the definition of TBI in this population.

## Results

**Figure 2:**
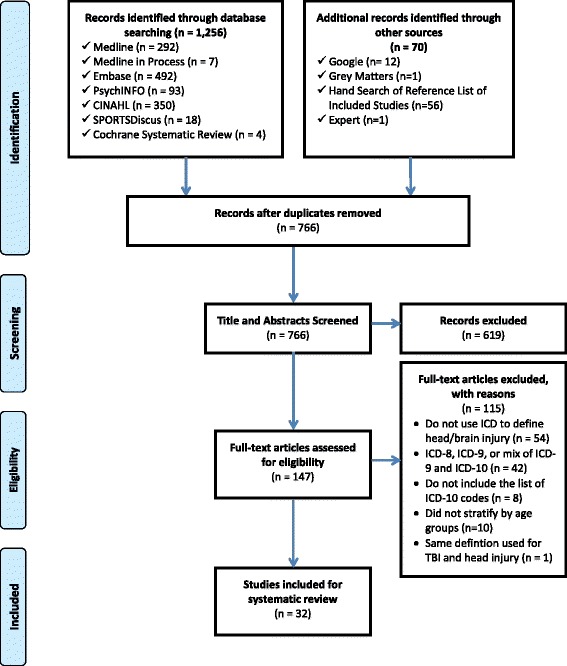
**PRISMA study flow diagram.**

A total of 1,256 articles were identified through database search, 56 articles were identified through hand searching of the reference lists of included studies, and 14 reports were identified through Grey Matters, Google, and expert consultation. After duplicates were removed, 766 title and abstracts were screened. Of these, 147 full text articles were assessed for eligibility, resulting in 32 studies/reports for inclusion in this systematic review [1,33-63]. Please see Figure 2 for the PRISMA flow diagram of identification of articles for inclusion. Note that the peer-reviewed article by Harrison and colleagues in 2012 examined both head injury and TBI and provided a separate ICD-10 definition for both condition [41]. As such, this study is counted twice in the analysis but only once in the PRISMA study flow diagram. Quality assessment of ICD-10 codes revealed that only one study used validated codes [33].

The ICD-10 case definitions in identified articles and reports ranged from one that is broad such as S00 to S09 (injuries to the head), to ones that were narrower such as S06 only (intracranial injury) or S06.0 only (concussion) for mild TBI (mTBI). Slightly more than half of the articles examined TBI (n = 18) while the rest examined mTBI (n = 1), severe TBI (n = 1), head injury (n = 11), and abusive head trauma (n = 2). Identified articles by age of the target population included 19 years and under (n = 9), 25 years and under (n = 3), 16 years and older (n = 1), and across the lifespan (i.e., 0 years and older; n = 20). Twenty-five articles examined incidence and trends and 8 articles examined TBI related deaths. Of all identified articles/reports, only five specifically examined children and youth aged 19 years or under with TBI. Two of these articles examined TBI related deaths and three examined incidence and trends. Please see Table 1 for a summary of all included articles and reports in this review. Reported incidence of TBI ranged from 45 per 100,000 to 375 per 100,000 while reported incidence of head injury ranged from 15 per 100,000 to 1835 per 100,000 in the children and youth population among articles identified in this review. Please see Table 1 for the range of incidence and number of new cases for each study, if reported.

**Table 1:** **Summary of identified peer-reviewed articles and grey literature reports**

**Article**	**Source of Data**	**Year of Studies**	**Location of Studies**	**Age of Population**	**Range of Incidence Rates**	**ICD-10 Codes Validated?**	**Purpose of Study/Codes**
**(Author, Year)**							
**mTBI**							
Peloso et al., 2004 [45]	Swedish Hospital Discharge Register	1987 – 2000 (data from 1997 – 2000 are based on ICD-10)	Sweden	0 – 65+ Years	Ages 0 – 20 years:	No	Incidence & Trends
					240 – 375 per 100,000		
**TBI**							
Andelic et al., 2008 [33]	Medical Records at Ulleval University Hospital	May 15, 2005 – May 14, 2006	Oslo, Norway	0 – 75+	Ages 0 – 19 Years	Yes (compared with CT scans)	Incidence & Trends
					50 – 200 per 100,000		
Colantonio et al., 2010 [37]	National Ambulatory Care Reporting System & Discharge Abstract Database	April 1, 2002 – March 31, 2007	Ontario, Canada	0 – 85+ Years	Ages 0 – 24 Years	No	Incidence & Trends
					111.6 – 375.5 per 100,000		
Harrison et al., 2012 [41]	Australian Institute of Health & Welfare National Hospital Morbidity Database	July 1, 2000 – June 30, 2006	Australia	15 – 24 Years	Ages 5 to 19 Years	No	Incidence & Trends
					183.6 per 100,000		
Koskinen & Alaranta, 2008 [43]	National Hospital Discharge Register of Finland	1991 – 2005	Finland	0 – 80+ Years	Ages 0 – 19 Years	No	Incidence & Trends
					50 – 120 per 100,000		
Puljala et al., 2012 [46]	Oulu University Hospital Discharge Register	1999 & 2007	Finland	0 – 75+ Years	-	No	Incidence & Trends
Shao et al., 2012 [47]	Wuhan Medical Care Center for Women and Children Discharge Data	2002 – 2011	China	0 – 17 Years	-	No	Incidence & Trends
Sills et al., 2005 [48]	Colorado Traumatic Brain Injury Surveillance System	1994 – 2002	Colorado, United States	0 – 36 Months	**-**	No	Death
Xia et al., 2012 [51]	Medical Records Database of Wuhan Medical Care Center for Women and Children	2002 – 2011	China	0 – 4 Years	-	No	Incidence & Trends
**Note:** This article is included in the TBI section even though the title is “pediatric health trauma” because the text stated that the case definition is a “TBI case definition”							
WellFlorida Council, 2007 [52]	State of Florida, Department of Health, CHARTS, Office of Vital Statistics	1999 – 2005	Florida	0 – 65+ Years	-	Unclear	Death
Thurman et al., 1995 [53]	_	_	United States	Entire Lifespan	-	Unclear	Incidence & trends
Faul et al., 2010 [22]	National Hospital Discharge Survey, National Hospital Ambulatory Medical Care Survey, and National Vital Statistics System	2002 – 2006	United States	0 – 75+ Years	-	Unclear	Death
Hubbard, 2012 [56]	New Mexico Bureau of Vital Records and Health Statistics, Hospital Inpatient Discharge Data	2007 – 2011	New Mexico	0 – 85+ Years	-	Unclear	Death
Nestman, 2009 [57]	Discharge Abstract Database, Vital Statistics Deaths Database	1995 – 2004	Nova Scotia, Canada	0 – 25 Years	-	Unclear	Incidence & Trends
		(data from 2001 – 2004 are based on ICD-10 codes)					
Socie et al., 2011 [58]	Ohio Death Certificate Files	2002 – 2009	Ohio	0 – 18 Years	-	Unclear	Death
SafeKIDS [60]	Discharge Abstract Database	1996 – 2005	Atlantic Canada	0 – 14 Years	Ages 0 – 14 Years:	Unclear	Incidence & trends
		(data from 2001 – 2005 are based on ICD-10 codes)			600 – 800 per 100,000		
The Victorian Neurotrauma Intiative, 2009 [61]	National Hospital Morbidity Database	2008	Australia	0 – 85+	-	Unclear	Incidence & trends
CDC, 2011 [62]	CDC multiple cause of death public use data files	Jan. 1997 – Dec. 2007	United States	0 – 85+	-	Unclear	Death
Australian Institute of Health and Welfare, 2007 [63]	National Hospital Morbidity Database	2004 – 2005	Australia	0 – 80+	-	Unclear	Incidence & trends
**Severe TBI**							
Andelic et al., 2012 [34]	Patient Registry at Four Hospitals	Jan. 2009 – Dec. 2010	Norway	16+ Years	-	No	Incidence & trends
**Head Injury**							
Barker-Collo et al., 2009 [35]	National Health Information Service (hospital morbidity data)	July 1, 1997 – June 30, 2004	New Zealand	0 – 85+ Years	Ages 0 – 19 Years (2003/04): 15 – 75 per 100,000	No	Incidence & trends
		(data from 1999/00 to 2003/04 are based on ICD-10 codes)					
Bener et al., 2009 [36]	Emergency Medical Services Registry	2001 – 2006	Qatar	0 – 65+ Years	Incidence:	No	Incidence & trends
					1.6 – 19.7 per 10,000 (0 – 19 Years)		
Crowe et al., 2009 [38]	Royal Children’s Hospital Emergency Department Database	2004	Melbourne, Australia	0 – 16 Years	Mild – Severe Head Injury:	No	Incidence & trends
					31 – 1835 per 100,000		
Deb, S. 1999 [38]	Accident and Emergency Department Case Register	April 1, 1996 – March 31,1997	South Wales, United Kingdom	0 – 65+ Years	-	No	Incidence & trends
Fujiwara et al., 2012 [40]	Discharge Abstract Database	2002 – 2007	Canada	0 – 23 Months	Ages 0 – 23 Months:	No	Incidence & trends
					1.5 – 18.0 per 100,000		
Harrison et al., 2012 [41]	Australian Institute of Health and Welfare National Hospital Morbidity Database	July 1, 2000 – June 30, 2006	Australia	15 – 24 Years	Ages 15 – 19 Years:	No	Incidence & trends
					623 per 100,000		
Kleiven et al., 2003 [41]	Swedish Hospital Discharge Register	1987 – 2000	Sweden	0 – 85+ Years	Ages 0 – 19 Years:	No	Incidence & trends
		(data from 1997 – 2000 are based on ICD-10 codes)			300 – 450 per 100,000		
Steudel et al., 2005 [49]	Federal Bureau of Statistics	1972 – 2000	Germany	0 – 90+ Years	-	No	Incidence & trends
		(data from 1998 to 2000 are based on ICD-10 codes)					
Tennant, 2005 [50]	Hospital Episodes Statistics from the Department of Health and National Statistics	2001/02 – 2002/03	England	0 – 75+ Years	Ages 0 – 15 Years:	No	Incidence & trends
					355.8 per 100,000		
Canadian Institute for Health Information, 2006 [55]	National Trauma Registry Minimal Data Set, National Trauma Registry Comprehensive Data Set, National Ambulatory Care Reporting System	1994/95 – 2003/04	Canada	0 – 60+ Years	Ages 0 – 19 Years:	Unclear	Incidence & trends
					62.5 – 132.9 per 100,000		
Ontario Injury Prevention Resource, 2012 [59]	IntelliHEALTH Database	2007/08 – 2009/10	Ontario, Canada	0 – 90+	Ages 0 – 19 Years (Resulting from Falls):	Unclear	Incidence & trends
					Emergency Department: 3414 – 408.7 per 100,000		
					Hospitalization: 7.8 – 38.6 per 100,000		
**Intentional Injury**							
Parks et al., 2011 [44]	National Centre for Health Statistics National Vital Statistics System	2003 – 2007	United States	0 – 4 Years	Rate of TBI with abuse head trauma:	No	Death
					0.76 per 100,000		
					Rate of TBI without abuse head trauma:		
					4.14 per 100,000		
Parks et al., 2012 [54]	-	-	-	0 – 4 Years	-	Unclear	Death

### S00 superficial injury of head

Six of 33 articles used the codes S00.0 (superficial injury of scalp), S00.7 (multiple superficial injuries of head), S00.8 (superficial injury of other parts of head), and S00.9 (superficial injury of head, part unspecified) and five articles also included the remaining S00 codes. All of these articles examined head injury and used the broad S00 – S09 codes for their case definition. None were children and youth specific. Please see Tables 2 and 3.

**Table 2:** **ICD-10 case definitions of peer-reviewed articles**

**ICD-10 Code**	**Article (Author, Year)**																			
	**Andelic et al., 2008 [** 33 **]**	**Andelic et al., 2012 [** 34 **]**	**Barker-Collo et al., 2009 [** 35 **]**	**Bener et al., 2009 [** 36 **]**	**Colantonio et al., 2010 [** 37 **]**	**Crowe et al., 2009 [** 39 **]**	**Deb, S. 1999 [** 38 **]**	**Fujiwara et al., 2012 [** 40 **]**	**Harrison et al., 2012 [** 41 **]** **(TBI Codes)**	**Harrison et al., 2012 [** 41 **]** **(Head Injury)**	**Kleiven et al., 2003 [** 42 **]**	**Koskinen & Alaranta, 2008 [** 43 **]**	**Parks et al., 2011 [** 44 **]**	**Peloso et al., 2004 [** 45 **]**	**Puljala et al., 2012 [** 46 **]**	**Shao et al., 2012 [** 47 **]**	**Sills et al., 2005 [** 48 **]**	**Steudel et al., 2005 [** 49 **]**	**Tennant, 2005 [** 50 **]**	**Xia et al., 2012 [** 51 **]**
**S00 Superficial Injury of Head**																				
S00.0			X	X			X			X									X	
S00.1			X	X						X									X	
S00.2			X	X						X									X	
S00.3			X	X						X									X	
S00.4			X	X						X									X	
S00.5			X	X						X									X	
S00.6			X	X			X			X									X	
S00.7			X	X			X			X									X	
S00.8			X	X			X			X									X	
S00.9			X	X			X			X									X	
**S01 Open Wound of Head**																				
S01.0			X	X			X			X							X		X	
S01.1			X	X						X						X	X		X	X
S01.2			X	X						X						X	X		X	X
S01.3			X	X						X						X	X		X	X
S01.4			X	X						X						X	X		X	X
S01.5			X	X						X						X	X		X	X
S01.7			X	X			X			X						X	X		X	X
S01.8			X	X			X			X						X	X		X	X
S01.9			X	X			X			X						X	X		X	X
**S02 Fracture of Skull and Facial Bones**																				
S02.0	X		X	X	X	X	X	X	X	X	X	X	X			X	X	X	X	X
S02.1	X		X	X	X	X	X	X	X	X	X	X	X			X	X	X	X	X
S02.2	X		X	X		X				X	X							X	X	X
S02.3	X		X	X	X	X				X	X					X	X	X	X	X
S02.4	X		X	X		X				X	X							X	X	
S02.5	X		X	X		X				X	X							X	X	
S02.6	X		X	X		X				X	X							X	X	
S02.7	X		X	X	X	X	X	X	X	X	X	X	X			X	X	X	X	X
S02.8	X		X	X	X	X	X	X	X	X	X	X	X			X	X	X	X	X
S02.9	X		X	X	X	X	X	X	X	X	X	X	X			X	X	X	X	X
**S03 Dislocation, Sprain, and Strain of Joints and Ligaments of Head**																				
S03.0			X	X						X									X	
S03.1			X	X						X									X	
S03.2			X	X						X									X	
S03.3			X	X						X									X	
S03.4			X	X						X									X	
S03.5			X	X		X				X									X	
**S04 Injury of Cranial Nerves**																				
S04.0			X	X	X		X			X			X			X			X	X
S04.1			X	X			X			X									X	
S04.2			X	X			X			X									X	
S04.3			X	X			X			X									X	
S04.4			X	X			X			X									X	
S04.5			X	X			X			X									X	
S04.6			X	X			X			X									X	
S04.7			X	X			X			X									X	
S04.8			X	X			X			X									X	
S04.9			X	X			X			X									X	
**S05 Injury of Eye and Orbit**																				
S05.1			X	X						X									X	
S05.2			X	X						X									X	
S05.3			X	X						X									X	
S05.4			X	X						X									X	
S05.5			X	X						X									X	
S05.6			X	X						X									X	
S05.7			X	X						X									X	
S05.8			X	X						X									X	
S05.9			X	X						X									X	
**S06 Intracranial Injury**																				
S06.0	X	X	X	X	X	X	X	X	X	X	X	X	X	X	X	X	X	X	X	X
S06.1	X	X	X	X	X	X	X	X	X	X	X	X	X		X	X		X	X	X
S06.2	X	X	X	X	X	X	X	X	X	X	X	X	X		X	X	X	X	X	X
S06.3	X	X	X	X	X	X	X	X	X	X	X	X	X		X	X	X	X	X	X
S06.4	X	X	X	X	X	X	X	X	X	X	X	X	X		X	X	X	X	X	X
S06.5	X	X	X	X	X	X	X	X	X	X	X	X	X		X	X	X	X	X	X
S06.6	X	X	X	X	X	X	X	X	X	X	X	X	X		X	X	X	X	X	X
S06.7	X	X	X	X	X	X	X	X	X	X	X	X	X		X	X	X	X	X	X
S06.8	X	X	X	X	X	X	X	X	X	X	X	X	X		X	X	X	X	X	X
S06.9	X	X	X	X	X	X	X	X	X	X	X	X	X		X	X	X	X	X	X
**S07 Crushing Injury of Head**																				
S07.0	X		X	X	X	X	X			X					X	X	X		X	
S07.1	X		X	X	X	X	X	X		X			X		X	X	X		X	
S07.8	X		X	X	X	X	X	X		X			X		X	X	X		X	
S07.9	X		X	X	X	X	X	X		X			X		X	X	X		X	
**S08 Traumatic Amputation of Part of Head**																				
S08.0			X	X		X	X			X									X	
S08.1			X	X		X				X									X	
S08.8			X	X		X	X			X									X	
S08.9			X	X		X	X			X									X	
**S09 Other and Unspecified Injuries of Head**																				
S09.0			X	X		X	X			X									X	
S09.1			X	X		X	X			X									X	
S09.2			X	X		X				X									X	
S09.7	X		X	X		X	X	X		X			X			X	X		X	X
S09.8	X		X	X		X	X	X		X			X			X	X		X	X
S09.9	X		X	X		X	X			X						X	X		X	X
**Injuries Involving Multiple Body Regions**																				
T01.0																X	X			
T02.0					X							X				X	X			
T04.0	X															X	X			
T06.0	X															X	X			
**T90 Sequelae of Injuries**																				
T90.0																				
T90.1					X											X	X			X
T90.2					X								X			X	X			X
T90.3					X															
T90.4					X											X	X			X
T90.5					X								X			X	X			X
T90.8					X								X			X	X			X
T90.9					X								X			X	X			X

**Table 3:** **ICD-10 case definitions of grey literature reports**

**ICD-10 Code**	**Article (Author, Year)**												
	**WellForida Council, 2007 [** 52 **]**	**Thurman et al., 1995 [** 53 **]**	**Faul et al., 2010 [** 22 **]**	**Parks et al., 2012 [** 54 **]**	**Canadian Institute for Health Information, 2006 [** 55 **]**	**Hubbard, 2012 [** 56 **]**	**Nestman, 2009 [** 57 **]**	**Socie et al., 2011 [** 58 **]**	**Ontario Injury Prevention Resource, 2012 [** 59 **]**	**SafeKIDS** [60]	**Victorian Neurotrauma Initiative, 2009 [** 61 **]**	**CDC, 2011** [62]	**Australian Institute of Health and Welfare, 2007 [** 63 **]**
**S00 Superficial Injury of Head**													
S00.0									X				
S00.1									X				
S00.2									X				
S00.3									X				
S00.4									X				
S00.5									X				
S00.6									X				
S00.7									X				
S00.8									X				
S00.9									X				
**S01 Open Wound of Head**													
S01.0	X		X			X			X			X	
S01.1	X		X			X	X	X	X			X	
S01.2	X		X			X	X	X	X			X	
S01.3	X		X			X	X	X	X			X	
S01.4	X		X			X	X	X	X			X	
S01.5	X		X			X	X	X	X			X	
S01.7	X		X			X	X	X	X			X	
S01.8	X		X			X	X	X	X			X	
S01.9	X		X			X	X	X	X			X	
**S02 Fracture of Skull and Facial Bones**													
S02.0	X	X	X	X	X	X		X	X	X		X	X
S02.1	X	X	X	X	X	X	X	X	X	X		X	X
S02.2									X				
S02.3	X		X			X	X	X	X	X		X	
S02.4									X				
S02.5									X				
S02.6									X				
S02.7	X	X	X	X	X	X	X	X	X	X		X	X
S02.8	X		X	X	X	X	X	X	X	X		X	X
S02.9	X	X	X	X	X	X	X	X	X	X		X	
**S03 Dislocation, Sprain, and Strain of Joints and Ligaments of Head**													
S03.0									X				
S03.1									X				
S03.2									X				
S03.3									X				
S03.4									X				
S03.5									X				
**S04 Injury of Cranial Nerves**													
S04.0	X		X	X		X	X	X	X	X		X	
S04.1									X				
S04.2									X				
S04.3									X				
S04.4									X				
S04.5									X				
S04.6									X				
S04.7									X				
S04.8									X				
S04.9									X				
**S05 Injury of Eye and Orbit**													
S05.1									X				
S05.2									X				
S05.3									X				
S05.4									X				
S05.5									X				
S05.6									X				
S05.7									X				
S05.8									X				
S05.9									X				
**S06 Intracranial Injury**													
S06.0	X	X	X	X	X	X		X	X	X	X	X	X
S06.1	X	X	X	X	X	X	X	X	X	X	X	X	
S06.2	X	X	X	X	X	X	X	X	X	X	X	X	X
S06.3	X	X	X	X	X	X	X	X	X	X	X	X	X
S06.4	X	X	X	X	X	X	X	X	X	X	X	X	X
S06.5	X	X	X	X	X	X	X	X	X	X	X	X	X
S06.6	X	X	X	X	X	X	X	X	X	X	X	X	X
S06.7	X	X	X	X		X	X	X	X	X	X	X	
S06.8	X	X	X	X	X	X	X	X	X	X	X	X	X
S06.9	X	X	X	X	X	X	X	X	X	X	X	X	X
**S07 Crushing Injury of Head**													
S07.0	X		X			X	X	X	X	X		X	
S07.1	X	X	X	X	X	X	X	X	X	X		X	
S07.8	X		X	X	X	X	X	X	X	X		X	
S07.9	X	X	X	X	X	X	X	X	X	X		X	
**S08 Traumatic Amputation of Part of Head**													
S08.0									X				
S08.1									X				
S08.8									X				
S08.9									X				
**S09 Other and Unspecified Injuries of Head**													
S09.0									X				
S09.1									X				
S09.2									X	X			
S09.7	X		X	X		X	X	X	X	X		X	X
S09.8	X		X	X		X	X	X	X			X	
S09.9	X		X	X		X	X	X	X	X		X	
**Injuries Involving Multiple Body Regions**													
T01.0	X		X			X		X				X	
T02.0	X		X		X	X		X				X	
T04.0	X		X			X		X				X	
T06.0	X	X	X			X		X				X	X
**T90 Sequelae of Injuries**													
T90.0													
T90.1	X		X			X	X	X				X	
T90.2	X		X	X		X	X	X				X	
T90.3													
T90.4	X		X			X	X	X		X		X	
T90.5	X		X	X		X	X	X		X		X	
T90.8	X		X	X		X	X	X		X		X	
T90.9	X		X	X		X	X	X		X		X	

### S01 open wound of head

ICD-10 code S01.0 (open wound of scalp) was included in 11 articles. Codes S01.1 (open wound of eyelid and periocular area), S01.2 (open wound of nose), S01.3 (open wound of ear), S01.4 (open wound of cheek and temporamandibular area), and S01.5 (open wound of lip and oral cavity) appeared in 14 of 33 articles while codes S01.7 (multiple open wounds of head), S01.8 (open wound of other parts of head), and S01.9 (open wound of head, part unspecified) appeared in 15 articles. Four out of 5 articles that specifically examined TBI in children and youth used S01 codes – one article used all S01 codes while 3 articles used S01.1 to S01.9. Two of these articles captured TBI related deaths and the other two examined incidence and trends. Please see Tables 2 and 3.

### S02 fracture of skull and facial bones

ICD-10 codes S02.1 (fracture of base of skull), S02.7 (multiple fractures involving skull and facial bones), and S02.8 (fracture of other skull and facial bones) appeared in the majority of articles (n = 29). Codes S02.0 (fracture of vault of skull) and S02.9 (fracture of skull and facial bones, part unspecified) were included in 28 of 33 articles and ICD-10 code S02.3 (fracture of orbital floor) was found in 21 of 33 articles. The remaining S02 codes: S02.2 (fracture of nasal bone), S02.4 (fracture of malar maxillary bones), S02.5 (fracture of tooth), and S02.6 (fracture of mandible), appeared in 10 to 11 case definitions. All five articles on TBI in children and youth used S02.0, S02.1, S02.3, S02.7, S02.8, and S02.9 and one also included S02.2 in their case definition. Please see Tables 2 and 3.

### S03 dislocation, sprain, and strain of joints and ligaments of head and S05 injury of eye and orbit

Five of the 33 articles used ICD-10 code S03 and S05. All five articles examined head injury and used all ICD-10 codes in the S00 to S09 range. None of these articles were children and youth specific. Please see Tables 2 and 3.

### S04 injury of cranial nerves

ICD-10 code S04.0 (injury of optic nerve and pathway) was included in 18 of 33 articles and the remaining S04 codes were only used by articles that examined head injury (n = 6). All but one children and youth specific TBI articles included the S04.0 code, two of which examined incidence and trends. Please see Tables 2 and 3.

### S06 intracranial injury

The majority of articles included in this review used S06 codes. Specifically, codes S06.0 (concussion), S06.2 (diffuse brain injury), S06.3 (focal brain injury), S06.4 (epidural hemorrhage), S06.5 (traumatic subdural hemorrhage), S06.6 (traumatic subarachnoid hemorrhage), S06.8 (other intracranial injuries), and S06.9 (intracranial injury, unspecified) were included in 32 articles. Code S06.1 (traumatic cerebral oedema) was used in 31 articles and S06.7 (intracranial injury with prolonged coma) was identified in 30 articles. All five articles that examined TBI in children and youth used S06.0 and S06.2 to S06.9 and four of these also included S06.1 in their case definition. Please see Tables 2 and 3.

### S07 crushing injury of head

Among the 33 identified articles in this review, 24 included codes S07.1 (crushing injury of skull) and S07.9 (crushing injury of head, part unspecified) in the case definition. ICD-10 code S07.8 (crushing injury of other parts of head) was identified in 23 articles and code S07.0 (crushing injury of face) was included in 19 articles. Four of the five articles that examined TBI in children and youth used all S07 codes in their definition of TBI in this population. Please see Tables 2 and 3.

### S08 traumatic amputation of part of head

ICD-10 codes S08.0 (avulsion of scalp), S08.8 (traumatic amputation of other parts of head), and S08.9 (traumatic amputation of unspecified part of head) were included in 7 articles while code S08.1 (traumatic amputation of ear) was included in 6 articles. All articles that used S08 codes examined head injury. Please see Tables 2 and 3.

### S09 other and unspecified injuries of head

The majority of articles in this review included codes S09.7 (multiple injuries of head; n = 22), S09.8 (other specified injuries of head; n = 20), and S09.9 (unspecified injury of head; n = 19). However, only seven articles included codes S09.0 (injury of blood vessels of head, not elsewhere classified), S09.1 (injury of muscle and tendon of head), and S09.2 (traumatic rupture of ear drum). All five articles that examined TBI in children and youth included codes S09.7 and S09.9, four of which also included S09.8 while one of these five articles also included S09.2. Please see Tables 2 and 3.

### Injuries involving multiple body regions

ICD-10 codes T02.0 (fractures involving head and neck) and T06.0 (injuries of brain and cranial nerves with injuries of nerves and spinal cord at neck level) were included in 10 of the 33 articles in this review. Only seven articles used code T01.0 (open wounds involving head and neck) and nine articles used T04.0 (crushing injuries involving head and neck). Of the five articles that specifically examined children and youth with TBI, three articles used all injuries involving multiple body regions codes. Please see Tables 2 and 3.

### T90 sequelae of injuries of head

Sequelae of injuries of head codes were used by less than half of the articles included in this review. Specifically, T90.5 (sequelae of intracranial injury), T90.8 (sequelae of other specified injuries of head), and T90.9 (sequelae of unspecified injury of head) were included in 13 articles. ICD-10 code T90.2 (sequelae of fracture of skull and facial bones) was included in 12 articles, T90.4 (sequelae of injury of eye and orbit) in 11 articles, T90.1 (sequelae of open wound of head) in 10 articles, and T90.3 (sequelae of injury of cranial nerves) in one article. None of the articles that examined head injury included sequelae of injury of head codes in their case definition however, all five articles on children and youth with TBI included codes T90.4, T90.5, T90.8, and T90.9, four of which also included codes T90.1 and T90.2. Please see Tables 2 and 3.

## Discussion

This paper systematically reviewed the literature to explore the range of ICD-10 codes used to identify children and youth with TBI. A total of 32 articles were identified and only five articles specifically examined children and youth with TBI. One article examined both head injury and TBI and provided separate case definitions for these conditions. Validation of codes in research utilizing administrative data is important, however, only one of the included articles in this review stated that the case definition was validated (through comparison with results from CT scans) [33]. It is critical that the full range of codes that could potentially serve to improve data quality and for the planning of both prevention and treatment programs are validated. Reported incidence varied substantially and is also observed in a review of TBI in the general population [24] and in a systematic review on the incidence of concussion in contact sports [28]. The rates of head injury were higher than the rate of brain injury in this review, however, it is not surprising as codes for head injury typically include all codes within the S00 to S09 spectrum. Also, it is known that the incidence of TBI or head injury often peaks in infants (0 to 4 years) and older adolescents (15 to 19 years) [28,64,65] and as such, rates are not comparable across age categories, even among the 19 years and under population. Finally, it should be noted that not all papers and reports included in this review reported incidence rates (some studies included the number of new cases but did not provide the population count and as such, it was not possible to calculate the rates).

Findings from this review showed that a broad range of ICD-10 codes were used to define TBI, however, there was overwhelming consensus on the use of S06 codes. Conversely, ICD-10 codes S00, S03, S05, and S08 were only included in articles that examined head injury and in particular, all articles that included these codes utilized the broad head injury definition of ICD-10 codes S00 to S09. A literature search for evidence failed to reveal any relationship between these codes and brain injury among children and youth.

ICD-10 S01 codes were included in four of five articles that examined TBI in children and youth. From the Canadian Institute for Health Information (CIHI), which provides information on the coding standards in Canada, “open wounds include animal bites, cuts, lacerations, avulsion of skin, and subcutaneous tissue and puncture wounds with or without penetrating foreign body. They do not include traumatic amputations or avulsions that involve deeper tissue” [66]. This suggests that, in a Canadian context, S01 codes do not necessarily capture TBI. Also, two of the articles that specifically examined the incidence and trends of TBI in children and youth [47,51] utilized the United States Centre for Disease Control and Prevention (CDC) ICD-10 definition for TBI, which is used to identify TBI related deaths [22,53]. This suggests that further research is warranted to inform the inclusion of S01 codes in the definition of TBI, especially for morbidity rather than mortality. However, inclusion of this code may be beneficial for the purpose of prevention, where it is preferred to overestimate rather than underestimate cases and where near misses are of interest.

The majority of articles that examined TBI and all articles on children and youth with TBI included ICD-10 codes S02.0, S02.1, S02.3, S02.7, S02.8, and S02.9 in their case definitions. Many studies have identified TBI among individuals with facial fractures [11,12,46,67-76] as well as an increased risk for fractures of the orbital floor (S02.3) [68,71,75], malar maxillary bones (S02.4) [68,71], and the mandible (S02.6) [71,74]. Previous research has shown that brain injury is one of the most common concomitant injuries among children with mandibular and mid-facial fractures [74]. Also, patients with two or more facial fractures were 3.8 times more likely to have a TBI [72] and those with skull and orbital fractures had a significantly higher rate of concussions [75]. In the ED, skull fractures and intracranial injuries commonly occur together in children under the age of two. In particular, children younger than 12 months are at a higher risk for behavioural changes and loss of consciousness after a skull fracture [76]. In injuries from bicycle crashes, the odds of having an orbital fracture was 24.4 times higher among those who suffered an intracranial injury and the odds of having a maxillary fracture was 135 times higher among those with an intracranial injury than those without [68]. Kraus et al. found that motorcycle riders with a skull fracture had odds of TBI that were more than 10 to 12 times greater than those with no skull fracture. Further, fracture of the mandible, orbit, and zygoma were significantly associated with TBI among those wearing a helmet while fracture of the maxilla and zygoma were significantly associated with TBI among those not wearing a helmet [71]. As such, ICD-10 codes S02.0, S02.1, S02.3, S02.4, S02.6, S02.7, S02.8, and S02.9 are recommended for inclusion in the definition of TBI in children and youth, as there is strong evidence for the association of TBI with these conditions.

Of the S04 codes, only S04.0 (injury of optic nerve and pathways) was used often by articles that examined TBI, including four out of the five articles on children and youth. The literature also supports a relationship between optic nerve injury and brain injury [77,78], including a systematic review article that found that optic nerve sheath hemorrhages are significantly more common in pediatric abusive head trauma [79]. While some articles have indicated that injury of cranial nerves can be caused by brain injury [80-82], there is currently a lack of consensus and evidence for the inclusion of S04.1 to S04.9 codes in the definition of TBI for surveillance work that requires a precise definition.

ICD-10 S07 codes were included in four of five articles on TBI with children and youth. Although it has been reported that crushing injuries result in less severe neurologic damage and better outcome due to the cranium’s flexibility and ability to absorb slowly applied forces [83], brain damage can occur if the forces exerted exceed the tolerance of the cranium [84-88]. Moreover, it has been stated that the pediatric brain is particularly vulnerable [13]. Therefore, it is recommended that the definition of TBI in this population include S07 codes, especially for prevention purposes.

Select S09 codes were found in a majority of articles, including all five articles on children and youth with TBI. From CIHI, a “final diagnosis of ‘head injury’ is classified as an intracranial injury (brain injury) when any of the following is documented within the encounter: altered state of awareness, altered cognition, altered mentation, altered state of consciousness, Glasgow Coma Scale score of 3 – 12” and when the “final diagnosis is recorded as ‘head injury’ without further specification, assign S09.9” [66]. However, these conditions may be difficult to detect in the pediatric population due to limited communication abilities. Also from CIHI, a S09.7 code can be used when “injuries are classified to more than one of the categories S02 – S09.2” [66]. Therefore, this suggests that, in a Canadian context, it is important to include the codes S09.7, S09.8, and S09.9 in the definition of TBI in children and youth. However, it is recommended that data analysis should run these codes separately to determine the number of records with these codes, as it has been suggested that the use of ICD-9 code 959.01, unspecified head injury, over time has resulted in misclassification and lower specificity of the code for TBI [87,89].

Three of the five articles on children and youth with TBI included the injuries involving multiple body region codes. ICD-10 codes T01.0, T02.0, T04.0, and T06.0 are used if there is an injury classifiable to codes S01.X, S02.X, S07.X, S04.X, and S06.X and an associated neck injury code [89]. Because there is evidence for the inclusion of ICD-10 codes S02.X, S04.0, S06.X, and S07.X in the definition of TBI, T02.0, T04.0, and T06.0 should also be included in this definition, as excluding these codes may result in omitting of patients that have both a head injury and neck injury.

Sequelae of injuries of the head codes, T90, were found in four articles on children and youth with TBI. It has been suggested that inclusion of sequelae codes allows for the capturing of patients that were missed during their first admission, however, should only be included if the data source allows for the linkage of patients across the continuum of care, as it may otherwise result in double counting of patients [24]. It is also important to recognize that the long-term effects of a TBI may have severe negative impact on the individual [2,90-92]. As such, it is important to capture children and youth suffering from late effects of injuries, which can also assist in assessing the burden of TBI on the healthcare system. Interestingly, none of the included articles in this review used post-concussion syndrome code, F07.2, even though post-concussion symptoms have been reported to be permanent and debilitating [93-95]. It should be noted that a major publication from CIHI in 2007 on the burden of neurological diseases, disorders, and injuries in Canada, included the code F07.2. However, it was excluded from this review because it presented information on head and brain injury as one condition, including its definition [65]. Therefore, it is suggested that the definition of TBI in children and youth include sequelae of injuries of head codes T90.2, T90.4, T90.5, T90.8, T90.9, and F07.2.

Finally, many studies have identified retinal hemorrhage as a predictor of inflicted TBI in infants and young children [96], which includes shaken baby syndrome [97-101] and abusive head trauma [79,102]. Shaken baby syndrome is a form of abusive head trauma and inflicted TBI [103], resulting in intracranial hemorrhage [104]. It has been stated that retinal hemorrhage is present in 50% to 100% of cases and often clinches the diagnosis of shaken baby syndrome [100]. It also predicts brain injury in shaken baby syndrome [99] and it has been reported that retinal hemorrhage can rarely occur without intracranial hemorrhage or cerebral edema [105,106]. A systematic review on the clinical and radiographic characteristics associated with abusive and non-abusive head trauma also revealed that retinal hemorrhage is significantly associated with abusive head trauma [30]. Interestingly, the paper and report included in this review by Parks and colleagues did not include retinal hemorrhage in their definition [44,54]. Instead, the authors identified abusive head trauma based on the presence of the CDC case definition for fatal TBI with specific ICD-10 cause of injury codes. Nevertheless, given the strong evidence of retinal hemorrhage and brain injury in infants and children, it is suggested that the definition of TBI in children and youth include the ICD-10 code H35.6 (retinal hemorrhage).

Limitations of this review include the small number of papers that specifically examined children and youth with TBI. Also, the case definitions used were often modeled after the CDC’s ICD-10 definition for TBI related deaths, even if the objective of the paper was to capture TBI related ED visits or acute care admissions [47,51,60]. The CDC ICD case definition, in particular, the unspecified head injury codes, is influenced in part by coding practices [107]. However, the role of coding practices in other countries on the case definition of TBI is unknown. Education and studies on coding can assist in improving the sensitivity and specificity of case definitions for TBI. It is acknowledged that studies that were included in this review only captured cases that were admitted to a healthcare facility. Many milder cases of TBI may not seek medical attention and as such, research using ICD-10 codes to define patients with TBI may include only a selective group of individuals that seek/require healthcare services. Further, this systematic review did not place a restriction on including publications that only discussed a TBI diagnosis as the primary diagnosis because it may exclude relevant articles. As such, some cases identified in the included articles may not have been admitted to a healthcare setting primarily due to a TBI. However, the goal of this systematic review is to explore the range of ICD-10 codes used to identify children and youth with TBI in healthcare administrative data rather than describing the population of children and youth. As such, the inclusion of patients that may not have a TBI as their primary diagnosis is preferred over eliminating potentially relevant articles that contain important information on the case definition for defining children and youth with TBI. Nonetheless, interpretations should be made with these limitations in mind.

Future research should include systematic reviews on the association of ICD-10 codes S00 to S09 with brain injury in order to more accurately assess the relationship between these conditions and TBI, which may also assist in determining the most appropriate definition of TBI in children and youth for research using healthcare administrative data. More importantly, studies assessing the validity and accuracy of case ascertainment in administrative data for identifying TBI in children and youth should be conducted. Data quality in the Discharge Abstract Database, which captures acute care admissions in Canada, has been assessed using chart re-abstraction and indicated good agreement for non-clinical variables, moderate to substantial agreement for the most responsible diagnoses (the diagnosis most responsible for the acute care length of stay), and good specificity of S02, S06 codes [108]. However, this information is not available for other ICD-10 codes explored in this review. It is important to have accurate numbers for surveillance activity, as underestimates have implications for planning of healthcare services for this population and influence allocation of resources. Therefore, continuous monitoring of coding practices is crucial and will facilitate improved definition of TBI in children and youth in healthcare administrative data. It is further recommended that studies run separate analyses with and without ICD-10 codes S09.7, S09.8, S09.9, and H35.6 to determine the extent to which these codes inflate the number of identified cases. It is acknowledged that the inclusion of these codes will decrease the specificity of the definition, however, from the perspective of prevention efforts, it preferred to overestimate rather than underestimate. Conversely, it may be preferable to omit these codes for surveillance activity focused on understanding the healthcare utilization of this population.

## Conclusion

To the best of our knowledge, this is the first recent paper to systematically review the literature to explore the range of ICD-10 codes to define TBI in children and youth aged 19 years and under specifically. This focus on children and youth provides the opportunity to address coding issues that are unique to this population, which has the potential to be undercounted due to reporting difficulties, especially among infants. This review and additional literature search on the ICD-10 codes suggest that the following codes may be included in the definition of TBI in this population – S02.0, S02.1, S02.3, S02.4, S02.6, S02.7, S02.8, S02.9, S04.0, S06, S07, S09.7, S09.8, S09.9, T02.0, T04.0, T06.0, T90.2, T90.4, T90.5, T90.8, T90.9, F07.2, and H35.6. It is proposed that research focusing on the healthcare utilization of this population may benefit from using this set of codes, as it is more specific, with evidence from the literature that demonstrate an association of these codes with TBI. However, a broad definition of head injury (S00 to S09), including the “injuries involving multiple body region” codes (T02.0, T04.0, and T06.0) and “sequelae of injury” codes (T90.2, T90.5, T90.8, T90.9, and F07.2) is recommended for prevention purposes, as previous research has suggested that some cases may be missed with using a conservative definition and that a broader definition may be warranted for this population, especially for prevention purposes. This review provides evidence for discussion on how best to use ICD codes for different goals. It also provides a baseline of research at a specific point in time as we move forward to optimally improve the use of codes in a more standard way internationally.

## Additional file

Additional file 1:
**Search strategy.**

## Abbreviations

CDC: Centers for disease prevention and control
ED: Emergency department
ICD: International classification of diseases
ICD-9: International classification of diseases version 9
ICD-10: International classification of diseases version 10
TBI: Traumatic brain injury
