# Supplementary material for: Defining pediatric traumatic brain injury using International Classification of Diseases Version 10 Codes: A systematic review
Source: BMC Neurol. 2015 Feb 4;15:7. doi: 10.1186/s12883-015-0259-7 (PMC4335539; doi:10.1186/s12883-015-0259-7)
Supplement: Additional file 1: — Search strategy. [file 12883_2015_259_MOESM1_ESM.docx]

**Additional File 1. Search strategy**

**Medline (1946 to February Week 2 2013)
Medline In-Process (February 19, 2013)**

Search strategy:

------------------------------------------------------------------------------------------------------------

1. [CONCEPT 1: ICD10]
2. "International Classification of Diseases"/
3. "international classification of diseas*".mp.
4. "ICD-10*".mp.
5. ICD10*.mp.
6. "ICD ten*".mp.
7. "ICD 10*".mp.
8. or/2-7
9. exp Health Services Research/
10. "administrat* data".mp.
11. "medical record*".mp.
12. "health information*".mp.
13. surveillance.mp.
14. (claim or claims).mp.
15. "hospital discharg*".mp.
16. coding.mp.
17. codes.mp.
18. "Clinical Coding"/
19. "Medical Records"/
20. or/9-19
21. validity.mp.
22. validation.mp.
23. "case defin*".mp.
24. algorithm*.mp.
25. agreement*.mp.
26. accuracy.mp.
27. sensitivity.mp.
28. specificity.mp.
29. "predictive value*".mp.
30. exp "Predictive Value of Tests"/
31. or/21-30
32. 20 and 31
33. 8 or 32
34. [CONCEPT 2: Head Injury]
35. exp Craniocerebral Trauma/
36. exp Cerebrovascular Trauma/
37. "axonal injur*".tw.
38. concuss*.tw.
39. "shaken baby".tw.
40. retinitis/
41. exp brain edema/
42. ((brain or cerebral* or intra-crani* or intracrani*) adj3 (edema or oedema or swell*)).tw.
43. ((brain* or capitis* or cerebr* or crani* or hemispher* or intercrani* or inter-crani* or intracrani* or intra-crani* or skull*) adj3 (contusion* or damag* or fractur* or injur* or trauma* or wound*)).tw.
44. ((brain* or cerebr* or crani* or hemispher* or intercrani* or inter-crani* or head or intracrani* or intra-crani*) adj3 (haematoma* or haemorrhag* or hematoma* or hemorrhag* or pressure or bleed*)).tw.
45. ((retin* or eye* or ear* or gum* or palate* or scalp* or tooth* or teeth* or periocular* or mandib* or temporomandib* or cheek* or lip* or head* or skull* or facial* or face* or occipital* or parietal* or temporal*) adj3 (fractur* or injur* or trauma* or damag*)).tw.
46. retinitis*.tw.
47. ((retin* or brain* or cerebr* or crani* or hemispher* or intercrani* or inter-crani* or head or intracrani* or intra-crani*) adj3 (haematoma* or haemorrhag* or hematoma* or hemorrhag* or pressure or bleed*)).tw.
48. tbi.tw.
49. mtbi.tw.
50. or/35-49
51. [CONCEPT 3:Children]
52. exp child/
53. exp infant/
54. adolescent/
55. Young Adult/
56. exp pediatrics/
57. Pediatric*.tw.
58. child, hospitalized/
59. (youth* or newborn* or child* or girl* or boy* or infant* or adolescent* or teen* or preschool* or "pre school*" or baby or babies or kid* or pediatr* or paediatr*).tw.
60. or/52-59
61. 33 and 50 and 60
62. limit 61 to yr="1992-current"

***************************************************************************

**Embase (1980 to 2013 Week 07)**

Search Strategy:

--------------------------------------------------------------------------------------

1. [CONCEPT1: ICD-10]
2. "international classification of diseases"/ or icd-10/ or icd-10-cm/ or icd-10-pcs/
3. "international classification of diseas*".mp.
4. "ICD-10*".mp.
5. ICD10*.mp.
6. "ICD ten*".mp.
7. "ICD 10*".mp.
8. or/2-7
9. exp health services research/
10. "administrat* data".mp.
11. "medical record*".mp.
12. "health information*".mp.
13. surveillance.mp.
14. (claim or claims).mp.
15. "hospital discharg*".mp.
16. coding.mp.
17. codes.mp.
18. coding/ or patient coding/ or "coding and classification"/
19. medical record/ or electronic medical record/
20. or/9-19
21. validity.mp.
22. validation.mp.
23. "case defin*".mp.
24. algorithm*.mp.
25. agreement*.mp.
26. accuracy.mp.
27. sensitivity.mp.
28. specificity.mp.
29. "predictive value*".mp.
30. exp predictive value/
31. or/21-30
32. 20 and 31
33. 8 or 32
34. [CONCEPT 2: brain injury]
35. exp head injury/ or exp brain injury/
36. exp cerebrovascular accident/
37. "axonal injur*".tw.
38. concuss*.tw.
39. "shaken baby".tw.
40. exp maxillofacial injury/
41. exp brain edema/
42. ((brain or cerebral* or intra-crani* or intracrani*) adj3 (edema or oedema or swell*)).tw.
43. ((brain* or capitis* or cerebr* or crani* or hemispher* or intercrani* or inter-crani* or intracrani* or intra-crani* or skull*) adj3 (contusion* or damag* or fractur* or injur* or trauma* or wound*)).tw.
44. ((brain* or cerebr* or crani* or hemispher* or intercrani* or inter-crani* or head or intracrani* or intra-crani*) adj3 (haematoma* or haemorrhag* or hematoma* or hemorrhag* or pressure or bleed*)).tw.
45. ((retin* or eye* or ear* or gum* or palate* or scalp* or tooth* or teeth* or periocular* or mandib* or temporomandib* or cheek* or lip* or head* or skull* or facial* or face* or occipital* or parietal* or temporal*) adj3 (fractur* or injur* or trauma* or damag*)).tw.
46. retinitis*.tw.
47. ((retin* or brain* or cerebr* or crani* or hemispher* or intercrani* or inter-crani* or head or intracrani* or intra-crani*) adj3 (haematoma* or haemorrhag* or hematoma* or hemorrhag* or pressure or bleed*)).tw.
48. tbi.tw.
49. mtbi.tw.
50. or/35-49
51. [CONCEPT 2: children]
52. child/
53. infant/
54. adolescent/
55. pediatric*.hw.
56. hospitalized child/
57. (youth* or newborn* or child* or girl* or boy* or infant* or adolescent* or teen* or preschool* or "pre school*" or baby or babies or kid* or pediatr* or paediatr*).tw.
58. exp pediatrics/
59. Pediatric ward/
60. Childhood injury/
61. Child abuse/
62. or/52-61
63. 33 and 50 and 62
64. limit 63 to yr="1992-current"

*******************************************************************************

**PsychINFO (1805 to February Week 3 2013)**

Search Strategy:

--------------------------------------------------------------------------------------------------

1. [CONCEPT 1: ICD10]
2. "international classification of diseases"/
3. "international classification of diseas*".mp.
4. "ICD-10*".mp.
5. ICD10*.mp.
6. "ICD ten*".mp.
7. "ICD 10*".mp.
8. or/2-7
9. "health services research".mp.
10. "administrat* data".mp.
11. "medical record*".mp.
12. "health information*".mp.
13. surveillance.mp.
14. (claim or claims).mp.
15. "hospital discharg*".mp.
16. coding.mp.
17. codes.mp.
18. "clinical coding".mp.
19. exp Medical Records/
20. or/9-19
21. validity.mp.
22. validation.mp.
23. "case defin*".mp.
24. algorithm*.mp.
25. agreement*.mp.
26. accuracy.mp.
27. sensitivity.mp.
28. specificity.mp.
29. "predictive value*".mp.
30. statistical validity/
31. or/21-30
32. 20 and 31
33. 8 or 32
34. [CONCEPT 2: brain injury]
35. exp Traumatic Brain Injury/ or exp Brain Damage/ or exp Head Injuries/ or exp Head Banging/
36. exp cerebrovascular disorders/
37. "axonal injur*".tw.
38. concuss*.tw.
39. "shaken baby".tw.
40. retinitis.mp.
41. "brain edema".mp.
42. ((brain or cerebral* or intra-crani* or intracrani*) adj3 (edema or oedema or swell*)).tw.
43. ((brain* or capitis* or cerebr* or crani* or hemispher* or intercrani* or inter-crani* or intracrani* or intra-crani* or skull*) adj3 (contusion* or damag* or fractur* or injur* or trauma* or wound*)).tw.
44. ((brain* or cerebr* or crani* or hemispher* or intercrani* or inter-crani* or head or intracrani* or intra-crani*) adj3 (haematoma* or haemorrhag* or hematoma* or hemorrhag* or pressure or bleed*)).tw.
45. ((retin* or eye* or ear* or gum* or palate* or scalp* or tooth* or teeth* or periocular* or mandib* or temporomandib* or cheek* or lip* or head* or skull* or facial* or face* or occipital* or parietal* or temporal*) adj3 (fractur* or injur* or trauma* or damag*)).tw.
46. retinitis*.tw.
47. ((retin* or brain* or cerebr* or crani* or hemispher* or intercrani* or inter-crani* or head or intracrani* or intra-crani*) adj3 (haematoma* or haemorrhag* or hematoma* or hemorrhag* or pressure or bleed*)).tw.
48. tbi.tw.
49. mtbi.tw.
50. or/35-49
51. [CONCEPT 3: children]
52. pediatric*.mp.
53. (youth* or newborn* or child* or girl* or boy* or infant* or adolescent* or teen* or preschool* or "pre school*" or baby or babies or kid* or pediatr* or paediatr*).mp.
54. or/52-53
55. 33 and 50 and 54
56. 33 and 50
57. limit 56 to (100 childhood or 120 neonatal or 140 infancy or 160 preschool age or 180 school age or 200 adolescence or 320 young adulthood )
58. 55 or 57
59. limit 58 to yr="1992-current"

******************************************************************************

**CINAHL (1981 to Present)**

Search Strategy:

---------------------------------------------------------------------------------------------

1. [CONCEPT 1]
2. (MH “International Classification of Diseases”)
3. TX “International Classification of Diseas*)
4. TX “ICD-10*”
5. TX ICD10*
6. TX “ICD ten*”
7. TX “ICD 10*”
8. S2 OR S3 OR S4 OR S5 OR S6 OR S7
9. (MH “Health Services Research +”)
10. TX “administrat* data”
11. TX “medical record*”
12. TX surveillance
13. TX “health information*”
14. TX (claim or claims)
15. TX “hospital discharg*”
16. TX coding
17. TX codes
18. (MH “Coding”)
19. (MH “Medical Records+”)
20. S9 OR S10 OR S11 OR S12 OR S13 OR S14 OR S15 OR S16 OR S17 OR S18 OR S19
21. TX validity
22. TX validation
23. TX “case defin*”
24. TX algorithm*
25. TX agreement*
26. TX accuracy
27. TX sensitivity
28. TX specificity
29. TX “predictive value*”
30. (MH “Predictive Value of Tests”)
31. (MH “Sensitivity and Specificity”)
32. (MH “Reliability and Validity+”)
33. S21 OR S22 OR S23 OR S24 OR S25 OR S26 OR S27 OR S28 OR S29 OR S30 OR S31 OR S32
34. S20 AND S33
35. S8 OR S34
36. [CONCEPT 2]
37. (MH “Head Injuries+”)
38. TX “axonal injur*”
39. TX concuss*
40. TX “shaken baby”
41. (MH “Retinitis+”)
42. (MH “Cerebral Edema+”) OR (MH “Brain Injuries+”)
43. TX ((brain or cerebral* or intracrani* or intracrani*) n3 (edema or oedema or swell*))
44. TX ((brain* or capitis* or cerebr* or crani* or hemispher* or intercrani* or inter-crani* or intracrani* or intra-crani* or skull*) n3 (contusion* or damag* or fractur* or injur* or trauma* or wound*))
45. TX ((brain* or cerebr* or crani* or hemispher* or intercrani* or inter-crani* or head or intracrani* or intra-crani*) n3 (haematoma* or haemorrhag* or hematoma* or hemorrhag* or pressure or bleed*))
46. TX ((retin* or eye* or ear* or gum* or palate* or scalp* or tooth* or teeth* or periocular* or mandib* or temporomandib* or cheek* or lip* or head* or skull* or facial* or face* or occipital* or parietal* or temporal*) n3 (fractur* or injur* or trauma* or damag*))
47. TX retinitis*
48. TX ((retin* or brain* or cerebr* or crani* or hemispher* or intercrani* or inter-crani* or head or intracrani* or intracrani*) n3 (haematoma* or haemorrhag* or hematoma* or hemorrhag* or pressure or bleed*))
49. TX tbi
50. TX mbti
51. S37 OR S38 OR S39 OR S40 OR S41 OR S42 OR S43 OR S44 OR S45 OR S46 OR S47 OR S48 OR S49 OR S50
52. [CONCEPT 3]
53. (MH “Child+”)
54. (MH “Adolescence+”)
55. (MH “Young Adult”)
56. (MH “Pediatrics+”)
57. MW Pediatric*
58. TX (youth* or newborn* or child* or girl* or boy* or infant* or adolescent* or teen* or preschool* or “pre school*” or baby or babies or kid* or pediatr* or paediatr*)
59. S53 OR S54 OR S55 OR S56 OR S57 OR S58
60. S35 AND S51 AND S59
61. S35 AND S51 (Limiters – Age Groups: Infant, Newborn: birth-1 month, Infant: 1-23 months, Child Preschool: 2-5 years, Child: 6-12 years, Adolescent: 13-18 years, All Infant, All Child)
62. S60 OR S61 (Limiters – Published Date from: 19920101-)

***************************************************************************

**SPORTDiscus (1980 to Present)**

Search Strategy:

--------------------------------------------------------------------------------------------------

1. TX “international classification of diseas*”
2. TX “ICD-10*”
3. TX “ICD10*”
4. TX “ICD ten*”
5. TX “ICD 10*”
6. (S1 OR S2 OR S3 OR S4 OR S5)
7. TX “medical record*”
8. TX “administrat* data”
9. TX “hospital discharg*”
10. TX health n3 research*
11. TX “health information*”
12. TX surveillance
13. TX (claim or claims)
14. TX coding
15. TX codes
16. S7 or S8 OR S9 OR S10 OR S11 OR S12 OR S13 OR S14 OR S15
17. TX validity
18. TX validation
19. TX “case defin*”
20. TX algorithm*
21. TX agreement*
22. TX accuracy
23. TX sensitivity
24. TX specificity
25. TX “predictive value*”
26. S17 OR S18 OR S19 OR S20 OR S21 OR S22 OR S23 OR S24 OR S25
27. S16 AND S26
28. S6 OR S27
29. TX “axonal injur*”
30. TX concuss*
31. TX “shaken baby”
32. TX ((brain or cerebral* or intra-crani* or intracrani*) n3 (edema or oedema or swell*))
33. TX ((brain* or capitis* or cerebr* or crani* or hemispher* or intercrani* or inter-crani* or intracrani* or intra-crani* or skull*) n3 (contusion* or damag* or fractur* or injur* or trauma* or wound*))
34. TX ((brain* or cerebr* or crani* or hemisphere* or intercrani* or inter-crani* or head or intracrani* or intra-crani*) n3 (haematoma* or haemorrhag* or hematoma* or hemorrhag* or pressure or bleed*))
35. TX ((retin* or eye* or ear* or gum* or palate* or scalp* or tooth* or teeth* or periocular* or mandib* or temporomandib* or cheek* or lip* or head* or skull* or facial* or face* or occipital* or parietal* or temporal*) n3 (fractur* or injur* or trauma* or damag*))
36. TX retinitis*
37. TX ((retin* or brain* or cerebr* or crani* or hemispher* or intercrani* or inter-crani* or head or intracrani* or intra-crani*) n3 (haematoma* or haemorrhag* or hematoma* or hemorrhag* or pressure or bleed*))
38. TX tbi
39. TX mbti
40. DE "BRAIN -- Wounds & injuries" OR DE "BRAIN -- Concussion" OR DE "BRAIN damage" OR DE "CHRONIC traumatic encephalopathy" OR DE "HEAD injuries"
41. S29 OR S30 OR S31 OR S32 OR S33 OR S34 OR S35 OR S36 OR S37 OR S38 OR S39 OR S40
42. S28 AND S41
43. DE "CHILDREN" OR DE "AIDS (Disease) & children" OR DE "CHILD dancers" OR DE "CHILD development" OR DE "DANCE for children" OR DE "DEAFBLIND children" OR DE "EXERCISE for children" OR DE "OUTDOOR recreation for children" OR DE "OVERWEIGHT children" OR DE "PHYSICAL fitness for children" OR DE "SCHOOL children" OR DE "SELF-defense for children" OR DE "VIDEO games & children" OR DE "CHILD nutrition" OR DE "CHILDREN -- Diseases" OR DE "CHILDREN -- Health" OR DE "PEDIATRICS" OR DE "SPORTS for children" OR DE "TEENAGERS" OR DE "YOUTH"
44. TX (youth* or newborn* or child* or girl* or boy* or infant* or adolescent* or teen* or preschool* or "pre school*" or baby or babies or kid* or pediatr* or paediatr*)
45. SU (youth* or newborn* or child* or girl* or boy* or infant* or adolescent* or teen* or preschool* or "pre school*" or baby or babies or kid* or pediatr* or paediatr*)
46. S43 OR S44 OR S45
47. S42 AND S46

**********************************************************************************

**Cochrane Database of Systematic Reviews (2005 to January 2013)**

Search Strategy:

---------------------------------------------------------------------------------------------------------

- 1. [CONCEPT 1: ICD10]
  2. "international classification of diseas*".ti,ab,kw.
  3. "ICD-10*".ti,ab,kw.
  4. ICD10*.ti,ab,kw.
  5. "ICD ten*".ti,ab,kw.
  6. "ICD 10*".ti,ab,kw.
  7. or/2-6
  8. "administrat* data".ti,ab,kw.
  9. "medical record*".ti,ab,kw.
  10. "health information*".ti,ab,kw.
  11. surveillance.ti,ab,kw.
  12. (claim or claims).ti,ab,kw.
  13. "hospital discharg*".ti,ab,kw.
  14. coding.ti,ab,kw.
  15. codes.ti,ab,kw.
  16. or/8-15
  17. validity.ti,ab,kw.
  18. validation.ti,ab,kw.
  19. "case defin*".ti,ab,kw.
  20. algorithm*.ti,ab,kw.
  21. agreement*.ti,ab,kw.
  22. accuracy.ti,ab,kw.
  23. sensitivity.ti,ab,kw.
  24. specificity.ti,ab,kw.
  25. "predictive value*".ti,ab,kw.
  26. or/17-25
  27. [CONCEPT 2: Head Injury]
  28. "axonal injur*".tw.
  29. concuss*.tw.
  30. "shaken baby".tw.
  31. ((brain or cerebral* or intra-crani* or intracrani*) adj3 (edema or oedema or swell*)).tw.
  32. ((brain* or capitis* or cerebr* or crani* or hemispher* or intercrani* or inter-crani* or intracrani* or intra-crani* or skull*) adj3 (contusion* or damag* or fractur* or injur* or trauma* or wound*)).tw.
  33. ((brain* or cerebr* or crani* or hemispher* or intercrani* or inter-crani* or head or intracrani* or intra-crani*) adj3 (haematoma* or haemorrhag* or hematoma* or hemorrhag* or pressure or bleed*)).tw.
  34. ((retin* or eye* or ear* or gum* or palate* or scalp* or tooth* or teeth* or periocular* or mandib* or temporomandib* or cheek* or lip* or head* or skull* or facial* or face* or occipital* or parietal* or temporal*) adj3 (fractur* or injur* or trauma* or damag*)).tw.
  35. retinitis*.tw.
  36. ((retin* or brain* or cerebr* or crani* or hemispher* or intercrani* or inter-crani* or head or intracrani* or intra-crani*) adj3 (haematoma* or haemorrhag* or hematoma* or hemorrhag* or pressure or bleed*)).tw.
  37. [tbi.tw](http://tbi.tw/).
  38. [mtbi.tw](http://mtbi.tw/).
  39. or/28-38
  40. [CONCEPT 3: child]
  41. pediatric*.tw.
  42. (youth* or newborn* or child* or girl* or boy* or infant* or adolescent* or teen* or preschool* or "pre school*" or baby or babies or kid* or pediatr* or paediatr*).tw.
  43. 41 or 42
  44. 16 and 26
  45. 7 or 44
  46. 39 and 43 and 45
  47. limit 46 to yr="1992-current"

***************************************************************************

**Google and Grey Matters**

Search Strategy:

--------------------------------------------------------------------------------------------

1. “traumatic brain injury”
2. “traumatic brain injury in children and youth”
3. “traumatic brain injury international classification of disease”
4. “traumatic brain injury in children and youth international classification of disease”
5. “head injury”
6. “head injury in children and youth”
7. “head injury international classification of disease”
8. “head injury in children and youth international classification of disease”
